# Comprehensive Analysis of the Transcriptome-Wide m6A Methylome in Endometrioid Ovarian Cancer

**DOI:** 10.3389/fonc.2022.844613

**Published:** 2022-02-23

**Authors:** Li Yang, Xin Chen, Xiang Qian, Jiejie Zhang, Meijuan Wu, Aijun Yu

**Affiliations:** ^1^ Department of Gynecological Oncology, The Cancer Hospital of the University of Chinese Academy of Sciences (Zhejiang Cancer Hospital), Institute of Basic Medicine and Cancer (IBMC), Chinese Academy of Sciences, Hangzhou, China; ^2^ Department of Traditional Chinese Medicine, The Cancer Hospital of the University of Chinese Academy of Sciences (Zhejiang Cancer Hospital), Institute of Basic Medicine and Cancer (IBMC), Chinese Academy of Sciences, Hangzhou, China; ^3^ Department of Pathology, The Cancer Hospital of the University of Chinese Academy of Sciences (Zhejiang Cancer Hospital), Institute of Basic Medicine and Cancer (IBMC), Chinese Academy of Sciences, Hangzhou, China

**Keywords:** endometrioid ovarian cancer, N6-methyladenosine, MeRIP-seq, METTL3, modification patterns

## Abstract

Emerging studies have revealed that N6-methyladenosine modification is involved in the development of various cancers. However, the m6A modification pattern of endometrioid ovarian cancer (EOC) has not been demonstrated. In the present study, high-throughput sequencing combined with methylated RNA immunoprecipitation (MeRIP-seq) and RNA sequencing were used to obtain the transcriptome-wide m6A modifications of endometrioid ovarian cancer for the first time. The roles of methyltransferase-like 3 (METTL3) in EOC cell line COV362 were explored. In total, 39,237 m6A-modified peaks related to 17,082 genes were identified in the EOC group, and 52,848 m6A peaks representing 19,349 genes were detected in endometriosis group. Functional enrichment analysis revealed that m6A enriched genes were associated with tight junctions, cell adhesion molecules, platinum drug resistance, adherens junction, and more. METTL3 knockdown in the COV362 cells significantly decreased cell proliferation, promoted cell apoptosis, and induced cell cycle arrest at the G0/G1 phase. Our study presented the transcriptome-wide m6A modifications of endometrioid ovarian cancer for the first time and revealed various differentially expressed genes with methylated m6A modifications. This study may provide new directions for in-depth research of the underlying molecular mechanisms and signaling pathways of EOC development and progression.

## Introduction

Ovarian cancer is the third most common cancer worldwide and it has the highest mortality rate in females. According to the Global Cancer Statistics, there were 313,959 new cases diagnosed and 207,252 women died of the disease in 2020 (1). Epithelial ovarian cancer is the most common type of ovarian cancer. Endometrioid ovarian cancer (EOC) accounts for 10-15% of epithelial ovarian cancer (2, 3). Ovarian endometriosis (OE) is associated with EOC and is commonly considered a direct precursor lesion to endometrioid and clear cell ovarian cancer types (4). Most of the patients with EOC are diagnosed at an early stage with good prognosis. However, some patients still have early recurrence and metastasis with poor prognosis.

N6-methyladenosine (m6A) is the most abundant RNA modification types which accounts for nearly 80% of all methylation modifications in eukaryotic cells (5). It has been reported that multiple protein complexes were involved in the metabolism of m6A RNA methylation. It is catalyzed by a multicomponent methyltransferase complex, called “writers” and is removed by demethylases called “erasers”. “Writers” are mainly composed of three subunits: METTL3 functions as a catalytic subunit, methyltransferase-like 14 (METTL14) functions as a structural subunit, and Wilms’ tumor 1-associated protein (WTAP) works as an adaptor molecule (6–10). The identification of m6A demethylase indicates that m6A methylation in RNA is reversible and dynamic. Only two m6A “erasers”, fat mass and obesity-associated protein (FTO) and α-ketoglutarate-dependent dioxygenase homolog 5 (ALKBH5) have been reported so far (11, 12). “Readers” serve as binding proteins so that m6A group can exhibit biological functions. They consist of proteins that contain a YT521-B homology (YTH) domain, including YTHDF1-3, and YTHDC1-2 and insulin-like growth factor 2 mRNA-binding proteins 1, 2, and 3 (IGF2BP1, IGF2BP2, IGF2BP3) (13). Some other m6A “readers” have also been identified, such as fragile X mental retardation 1 (FMR1) and eukaryotic initiation factor 3 (eIF3), and the list is still growing (14).

Evidences suggest that m6A methylation affects all most every aspect of RNA metabolism, for example, RNA structure, RNA stability, mRNA nuclear export, translation, mRNA decay and noncoding RNA and miroRNA processing (15, 16). Accumulating data have indicated that m6A participates in many fundamental biological processes, such as embryogenesis, neurogenesis, stress responses, and DNA damage responses (17). Furthermore, there are emerging studies that have revealed that altered m6A modification participates in the development of cancers (18).

The detection of m6A modification patterns at the transcriptome-wide level helps to better understand the potential biological effects of m6A modification in RNA. With the development of MeRIP-seq in 2012 (19, 20), the transcriptome-wide distribution of m6A of several cancers have been reported. However, the profiles of transcriptome-wide m6A distribution in many cancers are still unknown.

To further investigate the functions of m6A, in this study, the combination of MeRIP-seq and RNA sequencing was applied to acquire the transcriptome-wide m6A modifications of EOC for the first time. Furthermore, the roles and potential mechanisms of METTL3 were studied in EOC cells *in vitro*.

## Materials and Methods

### Clinical Specimen

Three pairs of EOC samples and endometriosis samples were collected at the time of surgery in Zhejiang cancer hospital. All tissues were histopathologically reviewed by two gynecological pathologists independently. Samples were stored at -80°C in separated centrifuge tubes before use. The current study was approved by the Institutional Review Board of Zhejiang cancer hospital. All the patients recruited in the study provided a written informed consent.

### MeRIP-Seq and RNA Sequencing

Trizol reagent (Invitrogen, CA, USA) was used to extract total RNA from each sample according to the instruction. Bioanalyzer 2100 and RNA 6000 Nano LabChip Kit (Agilent, CA, USA) were used to conduct total RNA quality control and quantity assessment based on the threshold of RIN (RNA integrity number) values > 7.0. Approximately more than 200 μg of total RNA was obtained to isolate Poly (A) mRNA with poly-T oligo attached magnetic beads (Invitrogen). Then the fragmented RNA of about 100-nt-long oligonucleotides was incubated with m6A-specific antibody (No. 202003, Synaptic Systems, Germany) in IP buffer solution(50 mM Tris-HCl, 750 mM NaCl, and 0.5% Igepal CA-630) and with BSA (0.5 μg μl^−1^) for 2h at 4°C. Then, the mixture was incubated with protein-A beads. Bound RNA was eluted using elution buffer containing 1 × IP buffer and 6.7mM m6A. Eluted RNA was precipitated by 75% ethanol solution. Final cDNA libraries of eluted m6A-containing fragments (IP) and untreated input control fragments (input) were prepared using dUTP method in accordance with a strand-specific library preparation. The average insert length of the paired-end libraries was ~100 ± 50 bp. Subsequently, the IP samples and input samples were submitted to conduct 150bp paired-end sequencing on the Illumina Novaseq™ 6000 platform (LC-BIO Bio, Hangzhou, China) according to the recommended protocol.

### Data Analysis

After reads that contained adaptor contamination, low quality bases, and undetermined bases were removed using Cutadapt and perl scripts in-house, the sequence quality was verified by Fastp. The high-quality clean reads were mapped to the genome of *homo sapiens* (Version: v96) with default parameters using HISAT2 software. The mapping reads were used for peaking calling by R package exomePeak, while identified m6A peaks with bed or bam formats were visualized using IGV software (http://www.igv.org/). MEME and HOMER were used for *de novo* and known motif finding followed by localization of the motif involving peak summit by perl scripts in-house. Called peaks annotation and intersection with gene architecture were conducted using ChIPseeker. Next, expression level for all mRNAs from input libraries were calculated as FPKM (FPKM = [total_exon_fragments/mapped_reads (millions) × exon_length (kB)]) using StringTie. The differentially expressed peaks or mRNAs were identified with log_2_ (fold change) > 1 or log_2_ (fold change) < -1 and *P* value < 0.05 by R package edgeR.

### Cell Culture

Human EOC cell line COV362 was cultured in Dulbecco’s modified Eagle’s medium (DMEM, Hyclone, Tauranga, New Zealand) containing 10% fetal bovine serum (FBS, Gibco, NY, USA), 100 U/ml penicillin and 100 ug/ml streptomycin (Invitrogen, CA, USA) at 37°C incubator with a humidified atmosphere with 5% CO_2_.

### SiRNA Transfection

METTL3 down-regulation was achieved using sequence-specific small interfering RNA (siRNA). METTL3 siRNA and negative control was from GenePharma (Shanghai, China). COV362 cells grown in 6-well plates were transfected with 1 ug/ml sequence-specific siRNA or vector siRNA prepared in a mix with lipo3000 reagent and DMEM containing 10% serum. The cells were then cultured for 6 hours before use.

### Real Time PCR

Real time PCR was performed to detect whether METTL3 was successfully knockdown by siRNA, as described previously (21). The primer pairs of METTL3 used were 5’-CCCTATGGGACCCTGACAG-3’ (forward primer) and 5’-CTGGTTGAAGCCTTGGGGAT-3’ (reverse primer), and the primer pairs of GAPDH were 5’-CGGAGTCAACGGATTTGGTCGTAT-3’ (forward primer) and 5’- AGCCTTCTCCATGGTGGTGAAGAC-3’ (reverse primer).

### The MTT Assay

We planted COV362 cells at a concentration of 1×10^5^/ml in 96-well plates. Then cells were incubated in 200µl medium which contains 20 µl MTT (5 mg/mL) at 37°C for 4 h. Next, we discarded the medium and added 150 µl dimethyl sulfoxide (DMSO) into each well. After shaking for 10 minutes, the absorbance at 570nm were measured by Microplate Reader (BIO-RAD550, Hercules, CA) in each well. The cell viability rate was calculated as experimental OD value/control OD value × 100%.

### The TUNEL Assay

We used 4% paraformaldehyde solution to fix cells at room temperature for 30 minutes. Equilibration buffer were added to cover samples and incubated for 10 minutes. Then, enough TUNEL reagent (Roche, Basel, Switzerland) were added to cover samples and incubated at 37°C for 60 minutes. DAPI solution (Beyotime, Shanghai, China) was added to stain the nuclei. At last, the samples were analyzed using a fluorescent microscope (Olympus, Tokyo, Japan).

### Cell Cycle Assay

Flow cytometry was performed using the PI single staining method to conduct cell cycle analysis, as described previously (21). In general, we collected cells and suspended them in 1×PBS and then we fixed the cells in ethanol at 4°C overnight. Next, the cells were centrifuged for 5 min at 2000 rpm and resuspended 1×PBS and were centrifuged again. The cells were mixed with 500 μL of propidium (Beyotime, Shanghai, China) and kept in the dark for 10 min. Then flow cytometry (BD Biosciences, CA, USA) was used to analyze the samples.

### Western Blotting

Western blotting analysis was used to detect the alterations of protein levels, as previously described (21). The primary antibodies used in the experiments were against METTL3 (Abcam, ab195352), Cytokeratin 8 (KRT8, Abcam, ab53280), FAS (Abcam, ab133619) and GAPDH (Abcam, ab9485). The corresponding IgG-HRP secondary antibody was from Abcam (ab205718). The bend density was analyzed by Image J software (NIH, USA). The experiments were done at least three independent times for final analyses.

### Statistical Methods

All values were expressed as means ± standard error of the mean (SEM). One-way analysis of variance (ANOVA) was used to evaluate significant of differences in relative METTL3 mRNA expression in COV362 cells, cell viability and cell cycle analysis among multiple groups (Control, Vector and cells with METTL3 siRNA), and Student’s *t*-test was performed to compare relative METTL3 and KRT8 mRNA expression between EOC and OE groups. *P* value less than 0.05 was considered statistically significant. We performed all statistical analyses using SPSS (version 22.0, SPSS Inc., Chicago, IL, USA).

## Results

### Transcriptome-Wide Detection of m6A Modifications in EOC

Three pairs of human EOC and endometriosis samples were selected for MeRIP-seq and RNA sequencing assays.

**Figure 1:**
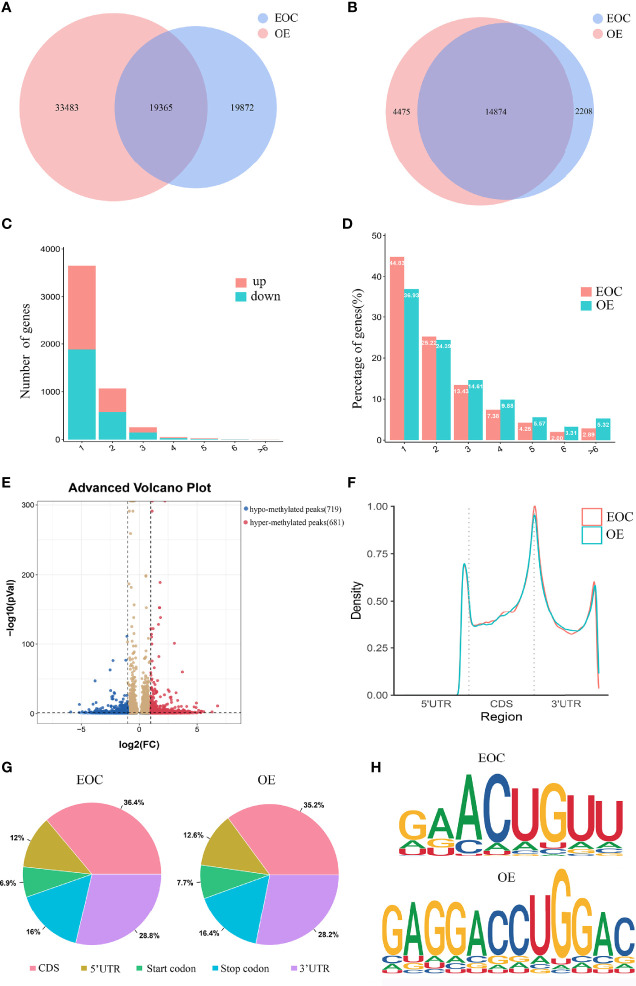
Characteristics of m6A distributions in EOC and OE. **(A)** Venn diagrams of overlaps of m6A peaks and **(B)** genes in EOC and OE tissues. **(C)** The distribution of differentially modified m6A peaks with significance per gene. **(D)** Number of peaks per gene in EOC and OE tissues. **(E)** Volcano plots showing the significant differential m6A peaks in EOC samples compared with OE samples. **(F)** The distribution and difference in the density of m6A peaks along all the transcripts in EOC and OE. **(G)** Pie charts showing the distributions of m6A peaks in EOC and OE tissues. **(H)** Representative m6A motifs enriched from the altered m6A peaks in EOC and OE groups, with *P*-values of 1*e^-166^ and 1*e^-247^, respectively.

A total of 39,237 m6A modified peaks were discovered in the EOC group, representing 17,082 genes. In the endometriosis group, 52,848 m6A peaks were identified, representing 19,349 genes. Among them, 19,365 m6A peaks and 14,874 genes were identified in both groups (**Figures 1A, B**). **Table 1** showed the top 30 altered m6A peaks in EOC group. The results indicate the significant difference in global m6A distribution in EOC and OE groups.

**Table 1:** The top 30 differently methylated m6A peaks (EOC vs OE).

Chromosome	Gene Name	Gene Id	Regulation	Fold Change	Start	End	*P* value
chr16	EIF3C	ENSG00000184110	up	6.77	28711721	28712060	0.00
chr18	AC105105	ENSG00000276403	up	6.29	58477131	58477425	0.00
chr8	AC100812	ENSG00000272192	up	5.57	65842781	65842955	0.00
chr22	PRAME	ENSG00000185686	up	5.39	22559031	22559241	0.00
chr19	DNM2	ENSG00000079805	up	5.34	10755219	10759693	0.00
chr20	GATA5	ENSG00000130700	up	5.32	62473503	62475102	0.03
chr12	HSPA8P14	ENSG00000257539	up	5.29	111384576	111384724	0.02
chr1	GATAD2B	ENSG00000143614	up	5.13	153852106	153852492	0.00
chr5	TMEM171	ENSG00000157111	up	5.04	73120688	73123386	0.03
chr1	HIST2H3A	ENSG00000203852	up	4.94	149854193	149860230	0.00
chr11	RNH1	ENSG00000023191	up	4.91	502587	502707	0.00
chr1	NBPF26	ENSG00000273136	up	4.91	120830503	120830563	0.03
chr8	AC107959	ENSG00000284956	up	4.81	23084385	23084532	0.01
chr12	OR7E47P	ENSG00000257542	up	4.74	52092513	52104297	0.00
chr14	SLC25A21	ENSG00000183032	up	4.62	36678341	36678580	0.01
chr1	FLG	ENSG00000143631	down	-5.91	152314711	152315402	0.00
chr8	FAM66E	ENSG00000225725	down	-5.17	8008396	8008546	0.02
chr16	CES1	ENSG00000198848	down	-5.16	55802851	55806387	0.00
chr1	SRGAP2	ENSG00000266028	down	-4.91	206404975	206405095	0.00
chr16	WFDC1	ENSG00000103175	down	-4.67	84325885	84326155	0.02
chrX	WASIR1	ENSG00000185203	down	-4.64	156015354	156016779	0.00
chr22	AC245452	ENSG00000224086	down	-4.58	21955197	21955347	0.02
chr16	NOMO3	ENSG00000103226	down	-4.33	16287792	16288639	0.00
chr21	APP	ENSG00000142192	down	-4.26	25945713	25945922	0.00
chr15	CHRFAM7A	ENSG00000166664	down	-4.26	30357796	30357976	0.01
chr19	SLC27A1	ENSG00000130304	down	-4.15	17468769	17470576	0.01
chr2	COL4A3	ENSG00000169031	down	-4.15	227283821	227284324	0.01
chr12	CSRNP2	ENSG00000110925	down	-4.12	51083455	51083515	0.01
chr10	DOCK1	ENSG00000150760	down	-4.12	127137756	127137995	0.02
chr2	C1QL2	ENSG00000144119	down	-4.11	119156273	119156511	0.02

By analyzing the alteration in the number of m6A peaks per gene, we found that most genes had one m6A peak. Approximately 45% of genes in EOC harbored one m6A peak, and about 83% of genes had 1-3 m6A modified sites. (**Figures 1C, D**)

Then we compared the abundance of the m6A peaks between EOC and OE samples. As shown in **Figure 1E**, 681 hyper-methylated m6A sites were discovered in EOC group compared with OE group, and a total of 719 hypo-methylated m6A sites were found.

Next, we investigated the distribution of differentially methylated m6A sites in transcriptome-wide scale of EOC and OE tissues. Each transcript was divided into start codon, 5’-UTR, CDS, 3’-UTR and stop codon. **Figure 1F** showed the distribution of m6A peaks in the two groups according to their locations in RNA transcripts. In general, the m6A peaks were especially enriched in CDS, 3’UTR, and stop codon in both groups (**Figure 1F, G**). Enrichment analysis results revealed m6A RRACH (R represents for G/A; A for m6A; H for A/C/U) consensus sequences, which reinforced the authenticity of the data (**Figure 1H**).

### Alterations of m6A Peaks Are Related to Important Signaling Pathways

**Figure 2:**
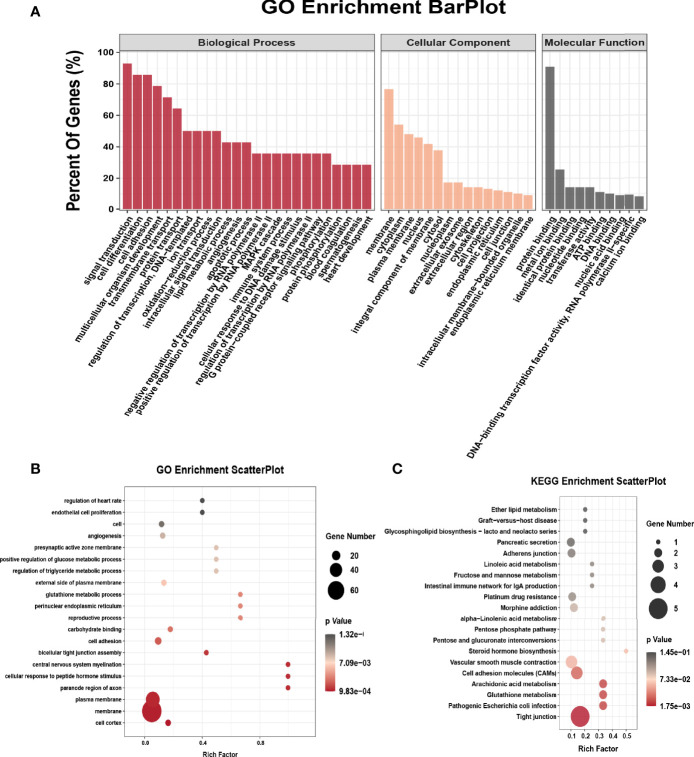
GO and KEGG pathway enrichment of hyper-methylated m6A genes. **(A)** Major enrichment and meaningful GO terms of hyper-methylated m6A genes in EOC. **(B)** The top 20 significant GO enrichment terms with m6A hypermethylation. **(C)** The top 20 significant KEGG pathways with m6A hypermethylation.

We performed GO analysis and KEGG analysis in hyper-methylated genes to evaluate the biological significance of m6A-enriched genes in EOC. GO analysis included three functional domains: biological process (BP), cellular component (CC) and molecular function (MF). The top 25 enriched BP terms, top 15 enriched CC terms and top 10 enriched MF terms of the hyper-methylated genes are shown in **Figure 2A**. The top 20 GO terms of genes with upregulated m6A peaks are shown in **Figure 2B**. **Figure 2C** shows that in KEGG analysis, hyper-methylated genes were associated with tight junction, pathogenic *Escherichia coli* infection, glutathione metabolism, cell adhesion molecules (CAMs), platinum drug resistance, adherens junction, etc.

### The Conjoint Analysis of MeRIP-Seq and RNA Sequencing

**Figure 3:**
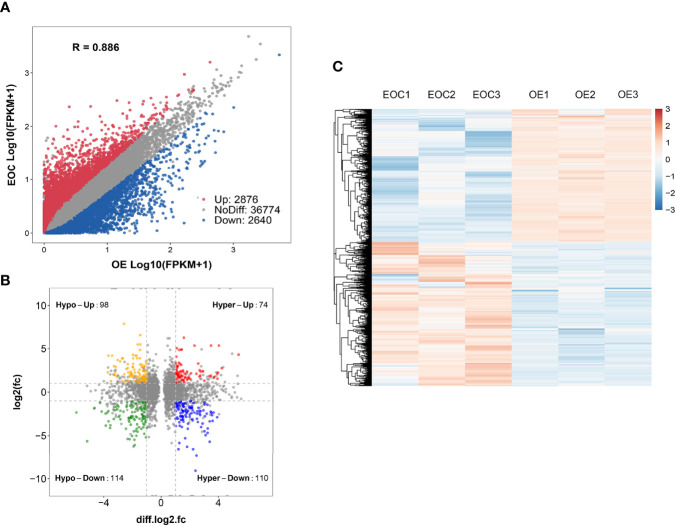
Conjoint analysis of MeRIP-seq and RNA sequencing data. **(A)** Scatter plot presenting the differentially expressed genes in EOC. **(B)** Four-quadrant plots presenting the distribution of genes with significant alterations in both the m6A modification and mRNA levels. **(C)** Heatmap plots exhibiting the differentially expressed genes of EOC and OE groups.

Compared to OE group, EOC group had 2876 significantly upregulated genes and 2640 significantly downregulated genes (**Figure 3A**).

**Figure 4:**
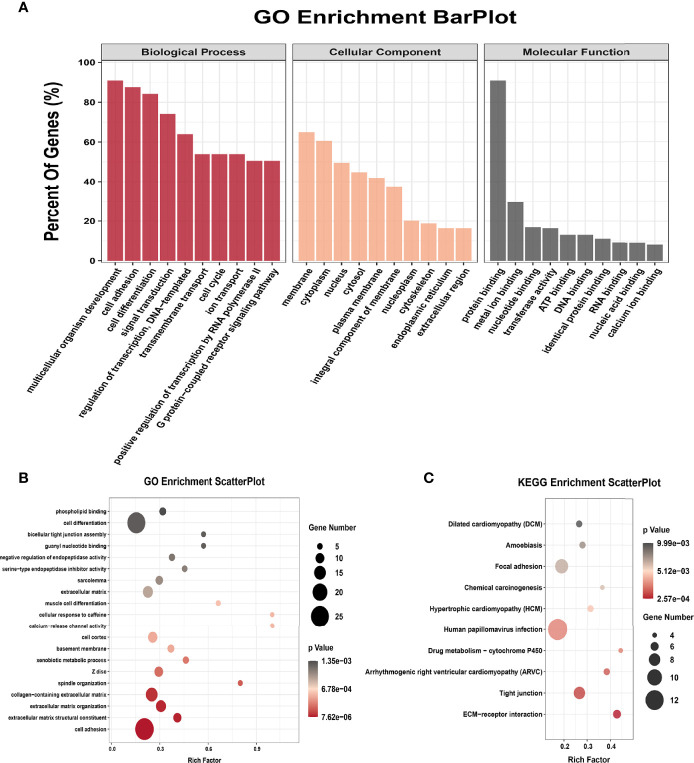
GO terms and KEGG pathways of genes with significant alterations in both the m6A modification and mRNA levels. **(A)** Major enrichment and meaningful GO terms of genes with significant alterations in both the m6A modification and mRNA levels. **(B)** The top 20 GO terms of genes with significant alterations in both the m6A modification and mRNA levels. **(C)** The top 10 KEGG pathways of genes with significant alterations in both the m6A modification and mRNA levels.

All genes were classified into 4 parts in conjoint analysis of MeRIP-seq and RNA sequencing data: a total of 184 hyper-methylated m6A peaks in which 74 mRNA transcripts were upregulated (hyper-up) and 110 mRNA transcripts were downregulated (hyper-down), and 212 hypo-methylated m6A peaks in 98 upregulated mRNA transcripts (hypo-up) or 114 down-regulated transcripts (hypo-down) (**Figure 3B**). A heatmap is created to investigate the differentially expression profiles of genes in EOC and OE (**Figure 3C**). GO analysis and KEGG pathway analysis were performed in those genes with differential expression and methylated m6A peaks synchronously. **Figure 4A** shows the top 10 terms of BPs, CCs, and MFs. **Figure 4B** shows the top 20 GO terms of these genes. KEGG pathway analysis showed that genes were enriched in human papillomavirus infection, focal adhesion, tight junction, and ECM−receptor interaction, etc. (**Figure 4C**).

### Functional Analysis of METTL3 in EOC Cell Line COV362

**Figure 5:**
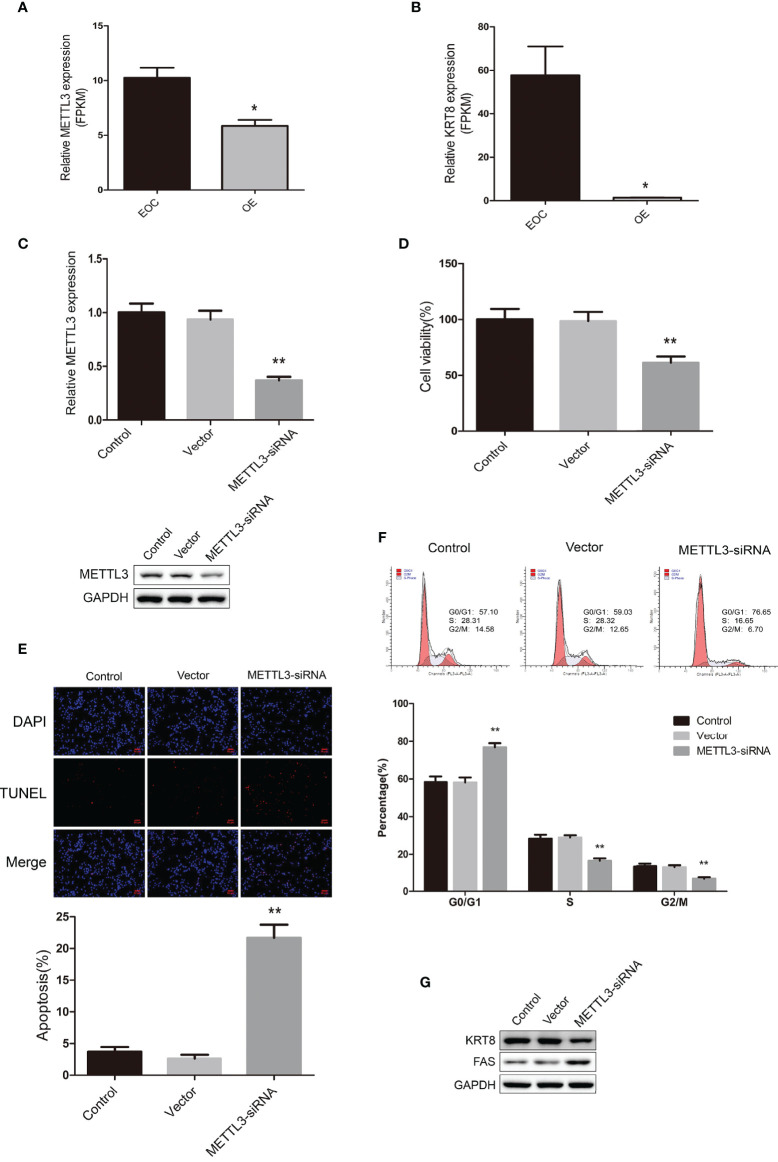
**(A)** The relative mRNA expression level of METTL3 in EOC and OE groups. **(B)** The relative mRNA expression level of KRT8 in EOC and OE groups. **(C)** Real-time PCR analysis and western blot analysis for METTL3 expression in COV362 untreated cells (Control), cells transfected with empty vector (Vector), and cells transfected with METTL3-siRNA (METTL3-siRNA). **(D)** MTT assay showing cell viability of Control, Vector and METTL3-siRNA cells. **(E)** The TUNEL assay showing the apoptosis rates of Control, Vector and METTL3-siRNA cells. **(F)** Cell cycle analysis of Control, Vector and METTL3-siRNA cells. **(G)** Western blot showing the expression levels of KRT8 and FAS in Control, Vector and METTL3-siRNA cells. * *P* < 0.05, ** Compared with the other groups, *P* < 0.01.

We further explored the data and found that METTL3 mRNA were highly expressed in the EOC group (**Figure 5A**). Also, the mRNA expression of KRT8 were significantly higher in the EOC group (**Figure 5B**). We then transfected the EOC cell line COV362 with METTL3 siRNA and successfully established a METTL3 knockdown cell line (**Figure 5C**). The MTT assays showed that the knockdown of METTL3 significantly led to the decreasing of cell proliferation (**Figure 5D**). The TUNEL assays revealed that knockdown of METTL3 increased cell apoptosis (**Figure 5E**). Furthermore, cell cycle analysis by flow cytometry showed that the percentage of cells in G0/G1 phase increased and S and G2/M phase decreased in cells with METTL3 knockdown (**Figure 5F**). Western blot data demonstrated that the expression level of KRT8 markedly decreased, and FAS increased in METTL3 knockdown cells (**Figure 5G**).

## Discussion

There are more than 170 modifications in RNA, and m6A is the most abundant modification in mRNA. M6A plays important roles in various aspects of RNA regulation, including RNA stability, splicing, degradation and translation. Increasing evidence suggests that m6A modification is involved in the tumorigenesis, proliferation, invasion, and progression of many kinds of malignancies (22, 23). M6A modification not only affects cancer functions such as cancer metabolism, cancer stem cell formation, epithelial–mesenchymal transition, but also participates in multiple signaling pathways (24). Although the transcriptome-wide m6A profiling of several cancers have been identified, the m6A methylation of EOC has not been reported. The present study identified the transcriptome-wide m6A distribution of EOC using a combination of MeRIP-seq and RNA sequencing for the first time.

In this study, we performed MeRIP-seq to determine the genome-wide profiling of m6A modification in EOC. Then the alterations of gene expression were also analyzed. Furthermore, GO analysis and KEGG pathway analysis were performed to evaluate the biological significance and pathophysiological roles of m6A-enriched genes and genes with differential expression and methylated m6A peaks synchronously in EOC by a conjoint analysis of MeRIP-seq and RNA-sequencing data. Additionally, we further explored the functions of METTL3 gene in EOC cells.

In the current study, we found that the difference in m6A modification between EOC and OE was significant. Briefly, 681 m6A peaks were upregulated and 719 m6A peaks were downregulated differentially in the EOC group. Furthermore, cancer-related biological pathways associated with hyper-methylated genes were significantly enriched. This suggests that m6A modification may contribute to EOC pathogenesis.

GO analysis revealed that cell adhesion-related hyper-methylated genes were enriched. There are four major groups of cell adhesion molecules: cadherins, integrins, selectins, and immunoglobulins (25). The integrin-mediated adhesion of epithelial cells to extracellular matrix proteins are lost and multiple signal transduction events are induced in the classic view of malignant transformation in the epithelium. This can be due to decreased E-cadherin expression resulting from transcriptional repression, its promoter methylation, or, uncommonly, a genetic or sporadic mutation (26–29). KEGG pathway analysis revealed that tight junctions are related to genes with up-methylated sites. Many researches have shown that tight junctions are closely related to the genesis and progression of many tumors. For instance, claudins, a group of membrane proteins that play a critical role in maintaining the proper function of epithelial tight junctions, were reported to participate in the carcinogenesis and metastasis of various cancers. Previous studies showed that claudin-1 overexpression is associated with the malignant behavior of colorectal cancer (30). Cytoplasmic expression of claudin-1 enhances the migratory ability of melanoma cells (31). Claudin-1 promotes the invasive ability of oral squamous cell carcinoma OSC-4 and NOS-2 cell lines through MT1-MMP and MMP-2 activation (32). E-cadherin expression was reduced by Claudin-1 *via* upregulating of ZEB-1 in colon cancer cells (33). Also, cell survival, invasion, and motility were increased in human ovarian surface epithelial cells with overexpression of claudin-3 and -4 increased (34). Hence, together with our data, we suggest that abnormal m6A methylation of genes participate in change of cell adhesion and tight junction, which may promote ovarian tumorigenesis and metastasis. Thus, modulating the m6A modification of genes in these pathways may provide new directions for the treatment of EOC.

Accumulating evidence has identified that METTL3 is implicated in human cancers either as an oncogene or a cancer suppressor in recent years. The controversial role of METTL3 in human cancer cells may be due to the different mechanisms of origin in various cancers. For instance, previous reports showed that METTL3 expression was significantly higher in hepatocellular carcinoma (HCC) than normal controls and it was correlated with shorter recurrence-free survival and overall survival (35). METTL3 knockout suppressed HCC oncogenicity and lung metastasis *in vivo* notably (36). It was demonstrated that suppressor of cytokine signaling 2 was the target of METTL3-mediated m6A modification and METTL3 repressed its expression through an m6A-YTHDF2-dependent pathway in liver cancer (36). Another study revealed that the epithelial-mesenchymal transition (EMT) was regulated by METTL3 in HCC cells through the methylating of CDS of Snail and then triggering polysome-mediated translation of Snail mRNA in cancer cells (37). It was reported that high expression of METTL3 was related with a poor prognosis in gastric cancer patients. In this study, Zinc finger MYM-type containing 1 (ZMYM1) was found to be a bona fide target of METTL3, which bound to and mediated the repression of E-cadherin promoter, thus driving the EMT program and leading to metastasis (38). Another study revealed that METTL3 level was elevated in colorectal cancer metastatic tissues and it was predictive of poor prognosis. SRY-box 2 (SOX2) was a downstream gene of METTL3, and the m6A of SOX2 transcripts was recognized by IGF2BP2 to prevent mRNA degradation (39). It was also reported that METTL3 can act as a tumor suppressor in some type of cancers. Liu et al. reported that about 70% of endometrial tumors exhibit reduced m6A levels than normal endometrium. The differences were likely due to a METTL14 mutation or reduced expression of METTL3, which promotes cell proliferation and the tumorigenicity of endometrial cancer through the activation of AKT signaling pathway (40).

A previous study showed that the expression of METTL3 and overall m6A level were elevated in EOC tissues, and the high expression of METTL3 was associated with poor malignancy and survival of EOC by mediating aberrant m6A modification (41). In our study, we found that METTL3 mRNA was highly expressed in the EOC group. Furthermore, the mRNA expression of KRT8 was significantly higher in the EOC group. We found that METTL3 knockdown in the EOC cell line significantly decreased cell proliferation, increased cell apoptosis, and induced cell cycle arrest at the G0/G1 phase. In addition, the expression level of KRT8 protein markedly decreased and FAS protein increased. As previous reports indicated KRT8 provide resistance to FAS-mediated apoptosis (42, 43), we deduced that knockdown of METTL3 may downregulate KRT8 expression, thus inducing FAS expression. However, whether METTL3 has an oncogenic role in EOC and is it mediated by KRT8 needs to be further explored. Also, more in-depth research should be done in the future to determine whether the function of METTL3 is related to its role as a methyltransferase.

In summary, we presented the transcriptome-wide m6A modifications of EOC for the first time using MeRIP-seq and discovered differentially expressed genes with various methylated m6A modifications. This study may provide new directions for in-depth research of the underlying molecular mechanisms and signaling pathways of EOC development and progression thus indicating new approaches for the treatment of EOC. Although some inhibitors of m6A methylation have been developed with promising effects, new regulators or inhibitors of m6A modifications should be further explored to provide novel therapeutic strategies for cancers.

## Data Availability Statement

The data presented in the study are deposited in the The Gene Expression Omnibus (GEO) repository. This data can be found here: https://www.ncbi.nlm.nih.gov/geo/query/acc.cgi?acc=GSE196748.

## Ethics Statement

The studies involving human participants were reviewed and approved by The Institutional Review Board of Zhejiang cancer hospital. The patients/participants provided their written informed consent to participate in this study.

## Author Contributions

AY and MW designed and supervised the whole study. LY and XC performed experiments and wrote the manuscript. XQ and JZ revised the manuscript. All authors contributed to the article and approved the submitted version.

## Funding

This work was supported by Basic Public Welfare Research Program of Zhejiang Province (LGF19H160011) and Medical and Health Science and Technology Project of Zhejiang Province (2019ZD001).

## Conflict of Interest

The authors declare that the research was conducted in the absence of any commercial or financial relationships that could be construed as a potential conflict of interest.

## Publisher’s Note

All claims expressed in this article are solely those of the authors and do not necessarily represent those of their affiliated organizations, or those of the publisher, the editors and the reviewers. Any product that may be evaluated in this article, or claim that may be made by its manufacturer, is not guaranteed or endorsed by the publisher.
